# The Evolution of Knockdown Resistance: Genotypic and Phenotypic Insights Into *Aedes aegypti* Insecticide Resistance

**DOI:** 10.1111/eva.70305

**Published:** 2026-07-23

**Authors:** Brook M. Jensen, Alden S. Estep, Silvie Huijben

**Affiliations:** ^1^ Center for Evolution and Medicine, School of Life Sciences Arizona State University Tempe Arizona USA; ^2^ United States Department of Agriculture, Agricultural Research Service Center for Medical, Agricultural and Veterinary Entomology Gainesville Florida USA; ^3^ Simon A. Levin Mathematical, Computational and Modeling Sciences Center Arizona State University Tempe Arizona USA

## Abstract

Insecticide resistance is a significant challenge for mosquito population control and disease prevention. This research investigated knockdown resistance (*kdr*) mutations V410L, V1016I, and F1534C in *Aedes aegypti* mosquitoes to address crucial gaps in understanding insecticide resistance evolution and management. Six common *kdr* genotypes were obtained through genetic crosses of three unique parental strains from the same location in Florida, USA. Through topical application of deltamethrin on both males and females, we quantified the resistance profiles of each genotype and sex. Triple homozygous mutants were over 52 times more resistant than the susceptible Rockefeller lab strain, with resistance ratios of the five other genotypes ranging from 8 to 19. We demonstrated that the resistant haplotypes **I** ^1016^‐**C** ^1534^ (on V410L mutant background) and **L** ^410^‐**I** ^1016^ (on F1534C mutant background) were incompletely recessive. Using these data in a simulation model, we show that the selective advantage is not constant but fluctuates within the window of selection. Selective advantage depended on the frequency of the resistance haplotype at the time of treatment, and both the level and dose of exposure. Finally, resistance profiles revealed that male mosquitoes had similar resistance profiles to females when sex and weight differences were accounted for, indicating that males could be used for resistance surveillance, which could enhance surveillance efforts. Overall, this study provides the first detailed quantification of dose–response curves and selection advantages for both sexes across multiple *kdr* genotypes. These results offer critical insights into the evolutionary dynamics of insecticide resistance and support the development of evidence‐based strategies for more sustainable mosquito control.

## 1 Introduction

Insecticides are a key tool for effective vector control (Bhatt et al. 2015). However, resistance has evolved against most classes of insecticides and is now observed across the globe, threatening public health and agriculture (Dusfour et al. 2019; Gould et al. 2018; Ranson et al. 2010; Reid and McKenzie 2016; World Health Organization 2018). Many well‐established methods depend heavily on insecticides, such as insecticide‐treated bed nets for malaria control or fogging to control the transmission of a wide range of arboviruses; as such, the availability of effective insecticides will remain critical. Therefore, insecticides should be used at levels that optimize mosquito control while minimizing selective pressure for resistance (Helps et al. 2017; Namias et al. 2021). Recent studies suggest that under certain conditions, such as lower doses of insecticides, the selection for resistance may be significantly reduced (Helps et al. 2017), similar to findings in pathogens and cancer cells (Huijben et al. 2013; Kouyos et al. 2014; Read et al. 2011; Zhang et al. 2017). To create efficient strategies for managing resistance in mosquito control, it is important to understand and quantify the circumstances under which selection exerts the greatest and weakest pressure for resistance evolution, which currently remains largely unknown.

Pyrethroids are one of the most commonly used insecticide classes worldwide due to their low toxicity to mammals, affordability, and rapid knockdown effects (World Health Organization 2005; Zaim et al. 2000). This widespread usage has led to the worldwide emergence of pyrethroid resistance in all three main disease‐transmitting mosquito genera: *Aedes*, *Anopheles*, and *Culex* (Ranson and Lissenden 2016; Rinkevich et al. 2013). Resistance can be caused by a variety of mechanisms, including target‐site, metabolic, cuticular, and behavioral (reviewed in Belinato and Martins 2016; Hemingway and Ranson 2000). Although pyrethroid resistance is often shaped by multiple interacting mechanisms, knockdown resistance (*kdr*) mutations in *Aedes aegypti* , the main vector of arboviruses such as dengue, Zika, and chikungunya, are among the most widespread and best‐characterized insecticide resistance mechanisms in mosquitoes. Specifically, *kdr* mutations V1016I and F1534C are broadly distributed. The V1016I mutation confers resistance to both types of pyrethroids only when in combination with one or more other *kdr* mutations (Chen et al. 2019; Du et al. 2013), and is commonly found in the Americas, with some occurrences in Africa (Chen et al. 2019; Kawada et al. 2016) and off the coast of Africa in Madeira (Seixas et al. 2017). The F1534C mutation confers resistance to type I pyrethroids (Hu et al. 2011) and possibly also type II pyrethroids (Fan and Scott 2020) and is widespread globally (Africa, the Americas, Asia, Europe) (Chen et al. 2019; Du et al. 2016; Kawada et al. 2016; Rajatileka et al. 2011; Seixas et al. 2017; Vera‐Maloof et al. 2015; Yanola et al. 2011). The frequency of F1534C has been increasing since it was first detected in Grand Cayman in 2008 (Harris et al. 2010), with populations in Brazil and Madeira at fixation (Granada et al. 2021; Seixas et al. 2017). The V1016I and F1534C mutations are in linkage disequilibrium (Brito et al. 2013; Vera‐Maloof et al. 2015) and appear to act synergistically (Brito et al. 2018), meaning they provide more resistance together than separately. A more recently discovered *kdr* mutation within *Ae. aegypti* is V410L, which confers resistance to both type I and type II pyrethroids (Haddi et al. 2017), and is also in linkage disequilibrium with V1016I and F1534C (Saavedra‐Rodriguez et al. 2018). The V410L mutation has been detected in various regions, including Brazil (Haddi et al. 2017; Melo Costa et al. 2020), Colombia (Granada et al. 2018; Silva et al. 2021), Mexico (Saavedra‐Rodriguez et al. 2018), and the USA (Estep et al. 2025; Kosinski et al. 2022; Mack et al. 2021; Wimmer et al. 2025). Despite mounting evidence of increasing and widespread pyrethroid resistance, pyrethroid usage in vector control persists due to the lack of effective and safe alternatives.

The Window of Selection (WoS), also previously called mutation‐selection window (MSW), has been used historically as a theoretical framework to understand the selective pressure acting upon resistance‐evolving pathogens such as antibiotic‐resistant bacteria (Drlica and Zhao 2007). Using detailed dose–response curves, it predicts the range of drug dosages between which selection for mutants is likely. The upper limit of the WoS refers to the mutant prevention concentration (MPC) and is the ideal target dose for resistance prevention and management, as it kills both susceptible and resistant organisms. The lower limit of the WoS is the dose at which there is no selection for resistance, as susceptible organisms survive these dosages at equal or better rates than resistant organisms (Drlica and Zhao 2007; South et al. 2020). The strength of selection within this WoS is unlikely to be constant either (Huijben et al. 2013; Negri et al. 2000; South et al. 2020). Decades after its initial use, this pathogen‐based framework was only recently introduced to insecticide resistance evolution in mosquitoes (South et al. 2020). With mosquitoes being diploid organisms, the WoS for mosquitoes is slightly more complicated than for pathogens, as the level of selection is additionally influenced by the level of dominance of the resistance mutation(s). To our knowledge, full dominance of an insecticide resistance allele has never been observed, including *kdr* mutations (Fan and Scott 2020; Soderlund 2008; Vera‐Maloof et al. 2020).

However, there is a lack of data on genotype‐specific dose–response curves to accurately measure the level of dominance and predict the selective advantage within the WoS for different insecticides, mosquito species, and resistance mutations. The only studies that collected data on this include *Anopheles gambiae* with an unspecified *kdr* mutation exposed to multiple permethrin‐treated nets (Corbel et al. 2004), *Culex quinquefasciatus* larvae with an unspecified resistance mechanism treated with permethrin dosages (Georghiou and Taylor 1987), and *Ae. aegypti* with *kdr* mutations V410L, V1016I, and F1534C treated with a permethrin‐based product (Permanone) in the field where permethrin dosages were quantified via gas chromatography (Hernandez et al. 2025). Two additional studies collected genotype‐specific response data for a single insecticide dose, which include *Ae. aegypti* with a suspected V1016I *kdr* mutation treated with permethrin (Saavedra‐Rodriguez et al. 2008), and *Ae. aegypti* with *kdr* mutations V410L, V1016I, and F1534C treated with permethrin, deltamethrin, malathion, pirimiphos‐methyl, or bendiocarb (Maiga et al. 2024). This paucity of genotype‐specific dose–response data is a major gap of knowledge, since the strength of selection across dosages within the WoS is essential to inform mathematical models and develop optimal resistance management strategies for mosquito control (South et al. 2020).

Resistance management strategies were first developed for infectious diseases, such as combination treatment for HIV (Moore and Chaisson 1999), leprosy (Noordeen 2016), and tuberculosis (reviewed in Kerantzas and Jacobs 2017), and much can be learned from these developments. However, important distinctions exist in the evolution of resistance between pathogens and vectors. In contrast to typical clinical treatment of pathogens, vector control is usually based on overall population management, rather than eradication. Therefore, the required relative *per capita* treatment rate for mosquitoes may be lower compared to pathogens. Additionally, as insecticides naturally degrade over time, remaining mosquito populations are subjected to varying concentrations below the initial application level. While drugs also have a half‐life in a human host, drug treatment is typically aimed at keeping drug plasma levels above a critical threshold until, ideally, most or all of the pathogens are eradicated. In contrast, this exposure change in vector control is often overlooked when estimating the influence of insecticide treatments on resistance evolution (South and Hastings 2018). An additional difference with applying this framework from pathogens to mosquitoes is that mosquitoes are sexually reproducing organisms. However, since males do not play an epidemiological role, their resistance status is rarely studied. Many insecticide‐based vector control strategies target predominantly female mosquitoes with host‐searching and post‐feeding resting behaviors, such as insecticide‐treated bed nets and indoor residual spraying. However, from the perspective of resistance evolution, males play a critical role, as half of the alleles in a population reside in males. Thus, detailed data on male resistance levels and exposure rates are critically needed to be incorporated into resistance evolution models.

To understand the resistance profiles of common *kdr* genotypes and their potential impact on resistance evolution in mosquitoes, we collected highly replicated dose–response data for male and female *Ae. aegypti* from six distinct genotypes and a wildtype susceptible laboratory strain, using topical application bioassays with the type II pyrethroid deltamethrin. We demonstrate the importance of genotype, sex, degree of dominance, insecticide coverage, and starting resistance frequency for the selective advantage and the time to 50% resistance haplotype frequency ($T_{50}$) for *kdr* mutations V410L, V1016I, and F1534C within the WoS. While this controlled experimental framework does not capture the full complexity of resistance evolution in natural populations, it allows the contribution of specific *kdr* genotypes to selection dynamics to be examined in isolation.

## 2 Materials and Methods

### 2.1 Mosquito Genotypes

Rockefeller (ROCK) strain of *Ae. aegypti* (obtained from the Center for Disease Control and Prevention) was used as a susceptible control. Three parental strains, fixed at either 410, 1534, or at all three loci 410, 1016, and 1534, were used to create genetic crosses. These strains were isolates from field collections in St. Augustine, Florida, in June 2016 and genotyped via PCR and melt curve analysis as described in (Estep et al. 2018). Here, we will refer to these strain isolates as **LL**‐VV‐FF, VV‐VV‐**CC**, and **LL**‐**II**‐**CC** with respect to *kdr* loci 410, 1016, and 1534, respectively, with the mutant alleles shown in bold. Three additional genotypes were generated as described below from genetic crosses: V**L**‐VV‐F**C**, V**L**‐V**I**‐**CC**, and **LL**‐V**I**‐F**C** (see Table S1).

### 2.2 Crossing of the Field‐Isolated Strains

The three homozygous genotypes (generations 12 through 17 since isolation from the field), originally isolated and established as previously described (Estep et al. 2025), were reared separately at 27°C ± 1.5°C at 60%–80% RH, with a 12:12 L:D regimen at USDA in Gainesville, Florida. Larvae were provided brewer's yeast and bovine liver powder in a 3:2 ratio until pupation. Pupae were placed in individual 10 mL test tubes. Once emerged, pupal exuviae (molts) were used for PCR and melt curve analysis to confirm the genotype for *kdr* loci 1016 (Saavedra‐Rodriguez et al. 2007) and 1534 (Yanola et al. 2011). Virgin females and males of the same or different genotypes were combined at a 5:1 female‐to‐male ratio following the crosses in Table S1 with a maximum of 1000 individuals per cage. Adults were provided with 10% sucrose solution *ad libitum*, offered excess human blood meals once a week (starting three‐to‐four days after emergence), and eggs were collected every few days. Eggs were shipped to Arizona State University (Tempe, AZ) in plastic bags lined with moist paper towels for subsequent rearing and exposures.

### 2.3 Rearing of Mosquitoes

Eggs were submerged in tap water and placed for 30 min in a vacuum‐sealed container. Larvae were kept at 27°C and 80% RH with a photoperiod of 12:12 L:D at a density of 150–200 larvae per liter in a 21 × 24 × 7 cm plastic container containing 1.5 L tap water and fed either ground cat food (Purina cat chow complete, first half of study) or fish food (Aqueon Cichlid fish food pellets, second half of study) *ad libitum*. Larval water was replaced every two‐to‐three days, and new food was added. Upon emergence, adults were fed 10% sucrose solution *ad libitum* and kept in a 24.5 × 24.5 × 24.5 cm mesh cage at a density of 400–1000 mosquitoes per cage.

### 2.4 Topical Application Bioassay

Topical application bioassay with deltamethrin (Pestanal, Sigma‐Aldrich) was performed to generate mass‐relativized dose–response data, following previously described methods (Jensen et al. 2022). In brief, for each genotype and sex, three‐to‐seven‐day‐old mosquitoes were briefly knocked down by cold by either placing aspirated mosquitoes in 50 mL falcon tubes under ice or by placing the cage in a 4°C fridge. Both methods were completed for 10 min or until all the mosquitoes were no longer flying/walking. Immobilized mosquitoes were transferred into 3–3.5 oz. plastic or paper cups held on ice, sorted by sex, and weighed in cohorts (per genotype and sex) of small batches (15–24 mosquitoes), medium batches (41–96 mosquitoes), or large batches (104–220 mosquitoes). After weighing, mosquitoes were immediately dosed. A single 0.5 μL drop of a gravimetrically prepared insecticide in acetone solution (or acetone only for the unexposed group) was applied to the ventral thorax of each mosquito using a Hamilton PB600‐1 repeating dispenser connected to a 25 μL blunt tip syringe. Mosquitoes were dosed in groups of 15–25 per cup. Following dosing, mosquitoes were transferred back to their cup, provided 10% sucrose, and kept under standard rearing conditions. Mortality was assessed 24 h post‐exposure following WHO guidelines (World Health Organization 2016b) by a researcher blind to dose. Tests were repeated if control mortality exceeded 20%. Exposures were completed on at least four separate dates and mosquito batches for each genotype and sex to avoid pseudoreplication. Exposure doses were performed at random for each genotype until 15 dose–response data points were obtained with mortality greater than 0% and less than 100% across a range of dosages.

### 2.5 Statistical Analysis

The deltamethrin exposure dose per mosquito was calculated by dividing the exposure dose of deltamethrin (ng) by the average weight of a mosquito in that cohort (mg). The Abbott's mortality correction was applied for all data where control mortality was greater than 0% (Abbott 1987). A generalized linear model was fitted to probit‐transformed mortality data and log‐transformed insecticide dosages using a quasibinomial distribution to account for potential dispersion. Model fit was evaluated using residual deviance, residual degrees of freedom, and dispersion parameters. This model was used to estimate the lethal dose to kill 1%, 50%, and 99% of the population (LD_01_, LD_50_, and LD_99_, respectively). The resistance ratio (RR) was calculated using the LD_50_ of the genotype of interest in comparison to the LD_50_ of a susceptible control genotype (ROCK). The LD_50_ and RR values were compared using Wald ratio tests with Bonferroni correction (Wheeler et al. 2006). The degree of dominance (D) was calculated using LD_50_ values and following the formula provided by (Stone 1968) for two different groups of genotypes: (1) **LL**‐VV‐FF, **LL**‐V**I**‐F**C**, and **LL**‐**II**‐**CC**, (2) VV‐VV‐**CC**, V**L**‐V**I**‐**CC**, and **LL**‐**II**‐**CC**. This first group contained the V1016I + F1534C haplotype on a V410L mutant background, hereafter referred to as the **I** ^1016^‐**C** ^1534^ haplotype; the second contained the V410L + V1016I haplotype on a F1534C mutant background, hereafter referred to as the **L** ^410^‐**I** ^1016^ haplotype. For each group of genotypes, hereafter referred to as populations, the upper bound of the WoS was determined by LD_99_ of the most resistant genotype (always **LL**‐**II**‐**CC**), and the lower bound was determined by LD_01_ of the most susceptible genotype of the genotypes of interest.

### 2.6 Simulations

Between the LD_99_ and LD_01_ of each population, the survival of each genotype was predicted at 200 equally spaced doses based on the probit regression model. For every dose and population, the resistant and susceptible haplotype frequency after exposure to the selected insecticide dose ($P\prime_{R}$ and $P\prime_{S}$, respectively) were calculated by equations (1) and (2):

(1) $$P\prime_{R}=\frac{\left[P_{\mathrm{RR}}\left(P^{e}w_{\mathrm{RR}}^{e}+P^{u}w_{\mathrm{RR}}^{u}\right)+\frac{1}{2}P_{\mathrm{SR}}\left(P^{e}w_{\mathrm{SR}}^{e}+P^{u}w_{\mathrm{SR}}^{u}\right)\right]}{\bar{W}}$$

(2) $$P\prime_{S}=\frac{\left[P_{\mathrm{SS}}\left(P^{e}w_{\mathrm{SS}}^{e}+P^{u}w_{\mathrm{SS}}^{u}\right)+\frac{1}{2}P_{\mathrm{SR}}\left(P^{e}w_{\mathrm{SR}}^{e}+P^{u}w_{\mathrm{SR}}^{u}\right)\right]}{\bar{W}}$$

where $P_{\mathrm{RR}},$ $P_{\mathrm{SR}},$ and $P_{\mathrm{SS}}$ are the starting proportion of the population that contain two, one, and zero copies of the resistant haplotype, respectively; $P^{e}$ and $P^{u}$ represent the proportion of the population that is exposed or unexposed to the insecticide dose, respectively; $w_{\mathrm{RR}}^{e}$, $w_{\mathrm{SR}}^{e}$, and $w_{\mathrm{SS}}^{e}$ represent the fitness (survival) of the genotypes at the selected insecticide exposure (e) dose; $w_{\mathrm{RR}}^{u}$, $w_{\mathrm{SR}}^{u}$, and $w_{\mathrm{SS}}^{u}$ represent the fitness (survival) of the genotypes that are unexposed (u) to the insecticide, and this value is assumed to be 1, indicating no fitness cost to the resistance haplotype in the absence of insecticide; $\bar{W}$ represents the mean fitness (survival probability) of the population after insecticide application and is represented by equation (3):

(3) $$\bar{W}=P_{\mathrm{RR}}\left(P^{e}w_{\mathrm{RR}}^{e}+P^{u}w_{\mathrm{RR}}^{u}\right)+P_{\mathrm{SR}}\left(P^{e}w_{\mathrm{SR}}^{e}+P^{u}w_{\mathrm{SR}}^{u}\right)+P_{\mathrm{SS}}\left(P^{e}w_{\mathrm{SS}}^{e}+P^{u}w_{\mathrm{SS}}^{u}\right)$$

Selective advantage (z) was then calculated for each dose following equation (4):

(4) $$z=\frac{P\prime_{R}}{P_{R}}-1$$

where $P_{R}$ is the starting resistant haplotype frequency, set to either 0.5, 0.01, or 0.0001, of a population in Hardy–Weinberg equilibrium. Equations (1), (2) and (4) are modified from South et al. (2020). The number of generations until the resistant haplotype made up 50% of the population ($T_{50}$) was calculated for all 200 exposure doses for the simulated populations that started with $P_{R}<0.5$. This was completed by equation (5):

(5) $$T_{50}=\min\left\{\;\text{generations}\;\right|P_{R}^{\prime }\geq 0.5\;\}$$

where equation (1) was used to calculate the updated resistant haplotype frequency ($P_{R}^{\prime }$) for each generation. After each iteration, the newly calculated $P_{R}^{\prime }$ replaced $P_{R}$ as the input for the next calculation. Each iteration represented one generation, and the loop continued until $P\prime_{R}\geq 0.5$. All statistical analyses and simulations were completed in R version 4.2.2 (R Core Team 2022).

## 3 Results

All genotypes tested, except Rockefeller (ROCK), were named according to their genotype at *kdr* loci 410, 1016, and 1534, respectively, with the mutant alleles shown in bold. These genotypes ranged from the fully susceptible reference strain (ROCK) to a triple homozygous resistant genotype for *kdr* mutations V410L, V1016I, and F1534C (**LL**‐**II**‐**CC**). On average, 574 mosquitoes were exposed to deltamethrin for each genotype and sex, in addition to mosquitoes in the unexposed groups. Each genotype‐by‐sex combination was tested with at least nine different concentrations of deltamethrin, overall ranging from 0.00075 to 9.69 ng per mg mosquito. Goodness‐of‐fit statistics for all probit models are provided in Table S2.

**FIGURE 1:**
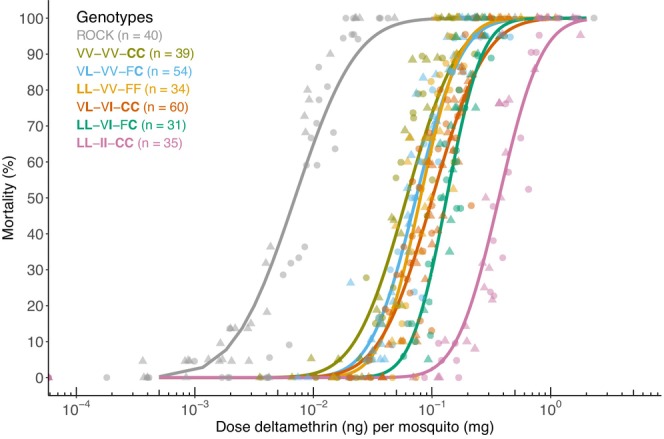
Dose–response curves of the seven *Ae. aegypti* genotypes (see legend) exposed to deltamethrin. Each data point shows the mortality of a group of 15–25 male (triangles) or female (circles) mosquitoes in the assay. The total number of groups plotted (males and females combined) is represented by the n‐values. The insecticide dose was calculated by ng deltamethrin per mean mg of mosquito mass in that group. The curved line shows the predicted mortality of each genotype's generalized linear model of both sexes combined with a quasibinomial distribution and probit link.

The triple homozygous mutant **LL**‐**II**‐**CC** was the most resistant genotype (Figure 1), being 52.5 times more resistant than ROCK (Table 1), and thus classified as ‘highly resistant’ following the World Health Organization's classification of resistance ratios (World Health Organization 2016a). The other two genotypes containing one copy of the triple mutant haplotype **L**‐**I**‐**C** (V**L**‐V**I**‐**CC** and **LL**‐V**I**‐F**C**), were also considered highly resistant for both sexes. The most susceptible *kdr* genotype tested was VV‐VV‐**CC**, at 8.7 times more resistant than ROCK, still considered moderately resistant. Each genotype's resistance ratio was significantly different from all other genotypes (Wald ratio test; *p*‐adj < 0.001), except for two comparisons: VV‐VV‐**CC** vs. V**L**‐VV‐F**C**, V**L**‐VV‐F**C** vs. **LL**‐VV‐FF.

**TABLE 1:** Resistance ratio (RR) values and 95% confidence intervals [95% CI] calculated as the ratio of LD_50_ values relative to those of Rockefeller mosquitoes of the same sex. Associated resistance levels are determined according to the World Health Organization (RR < 5 = susceptible; 5 < RR < 10 = moderately resistant; RR > 10 = highly resistant). Wald ratio tests with Bonferroni adjusted *p*‐values were used to determine significant differences between the genotypes; each unique group is indicated by a different letter (genotypes sharing a letter are not significantly different).

Genotype	RR [95% CI]	Resistance level	Significance groups
ROCK	1	Susceptible	*a*
VV‐VV‐CC	8.74 [7.14–10.66]	Moderately‐highly resistant	*b* *
VL‐VV‐FC	10.50 [8.80–12.53]	Moderately‐highly resistant	*b* *, *c* **
LL‐VV‐FF	11.42 [9.54–13.70]	Moderately‐highly resistant	*c* **
VL‐VI‐CC	14.43 [12.12–17.18]	Highly resistant	*d*
LL‐VI‐FC	19.02 [16.04–22.56]	Highly resistant	*e*
LL‐II‐CC	52.49 [43.49–63.34]	Highly resistant	*f*

* Wald *z* = 2.34; *p*‐adj = 0.41.

** Wald *z* = 1.29; *p*‐adj = 1.000.

The V410L mutation in isolation (genotype **LL**‐VV‐FF) conferred 1.3 times greater resistance against deltamethrin than the F1534C mutation in isolation (genotype VV‐VV‐**CC**). The same was seen when these mutations were in combination with other *kdr* mutations—genotype **LL**‐V**I**‐F**C** was 1.3 times more resistant than genotype V**L**‐V**I**‐**CC**. The only genotype with a double V1016I mutation (**LL**‐**II**‐**CC**) led to an increased resistance ratio value of 33.5–38.1 compared to the genotypes with one V1016I mutation (**LL**‐V**I**‐F**C** and V**L**‐V**I**‐**CC**), and genotype **LL**‐**II**‐**CC** was 2.8 times more resistant than the next most resistant genotype (**LL**‐V**I**‐F**C**). Therefore, the increased level of resistance observed in genotype **LL**‐**II**‐**CC** was likely due to the additional copy of the **L**‐**I**‐C haplotype and/or synergism between the *kdr* mutations.

**FIGURE 2:**
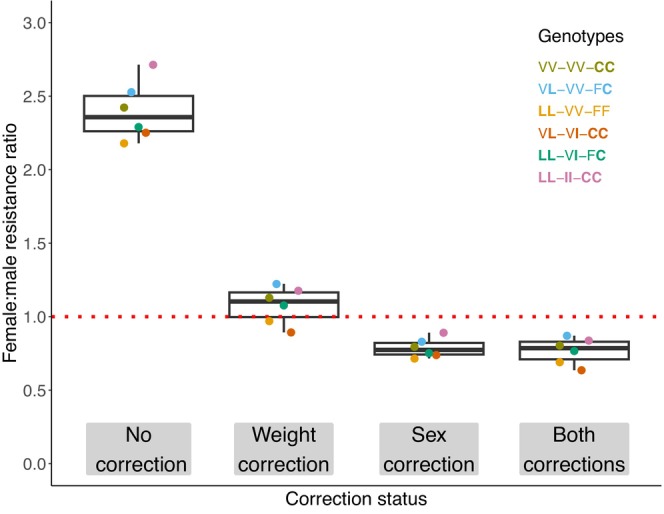
Female:male resistance ratio comparisons for deltamethrin. Weight correction considers the ng of deltamethrin per mg mosquito, while no weight correction uses the ng of deltamethrin per whole mosquito. Rockefeller mosquitoes of the same sex as the *kdr* genotypes are used for the sex correction, while only female Rockefeller mosquitoes are used for the calculations without sex corrections. Each correction status correlates to how the resistance ratios of each sex and genotypes were calculated (before being converted to a female:male ratio). Each point represents the ratio of the female to male RR values of a single genotype (see legend).

Male mosquitoes had higher mortality than female mosquitoes for the same insecticide concentration, although this difference was explained by their smaller body weight. After correcting for weight by calculating the lethal dose per mg body weight, there was no significant difference in the LD_50_ values between males and females of the same genotype for all genotypes (Wald ratio test, *p*‐adj ≥ 0.085 for all comparisons; Table S3). Furthermore, correcting for weight and/or using sex‐matched lethal dose calculations made a marked difference in the resistance ratios for females and males of the same genotype (Figure 2).

**FIGURE 3:**
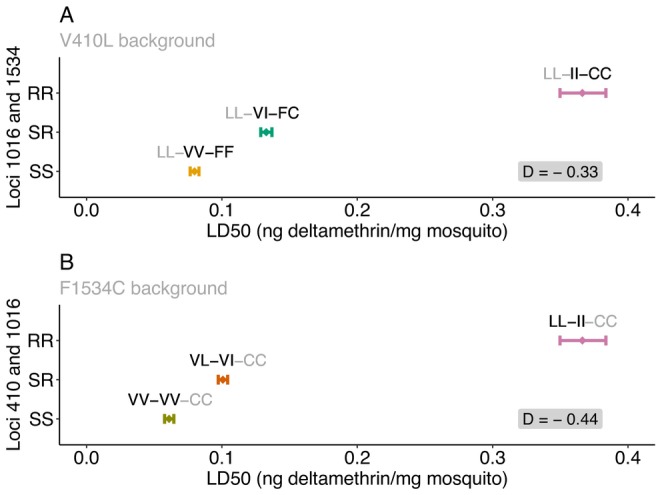
LD_50_ values of (A) homozygous susceptible, heterozygous, and homozygous resistant for the **I** ^1016^‐**C** ^1534^ haplotype, and (B) homozygous susceptible, heterozygous, and homozygous resistant for the **L** ^410^‐**I** ^1016^ haplotype. The background mutations for each are shown in gray. Error bars show 95% confidence intervals based on probit regression analysis, using dose–response data from both sexes. D‐values represent the degree of dominance based on the LD_50_ for the haplotype.

Both haplotypes **I** ^1016^‐**C** ^1534^ and **L** ^410^‐**I** ^1016^ were determined to be incompletely recessive (**I** ^1016^‐**C** ^1534^: D = −0.33, Figure 3A; **L** ^410^‐**I** ^1016^: D = −0.44, Figure 3B). Incomplete recessiveness still held true even when higher or lower than LD_50_ lethal dose values were used for calculating the degree of dominance (Figure S1). An interesting epistatic interaction was observed, where for the **I** ^1016^‐**C** ^1534^ haplotype the degree of dominance increased as the lethal dose increased, while the opposite was mostly seen for the **L** ^410^‐**I** ^1016^ haplotype. Thus, the degree of dominance varied depending on the specific combination of mutations and the concentration of insecticide applied. This dynamic suggests that the combined effects of the mutations are not simply additive but are influenced by complex interactions among genetic factors and environmental pressures.

**FIGURE 4:**
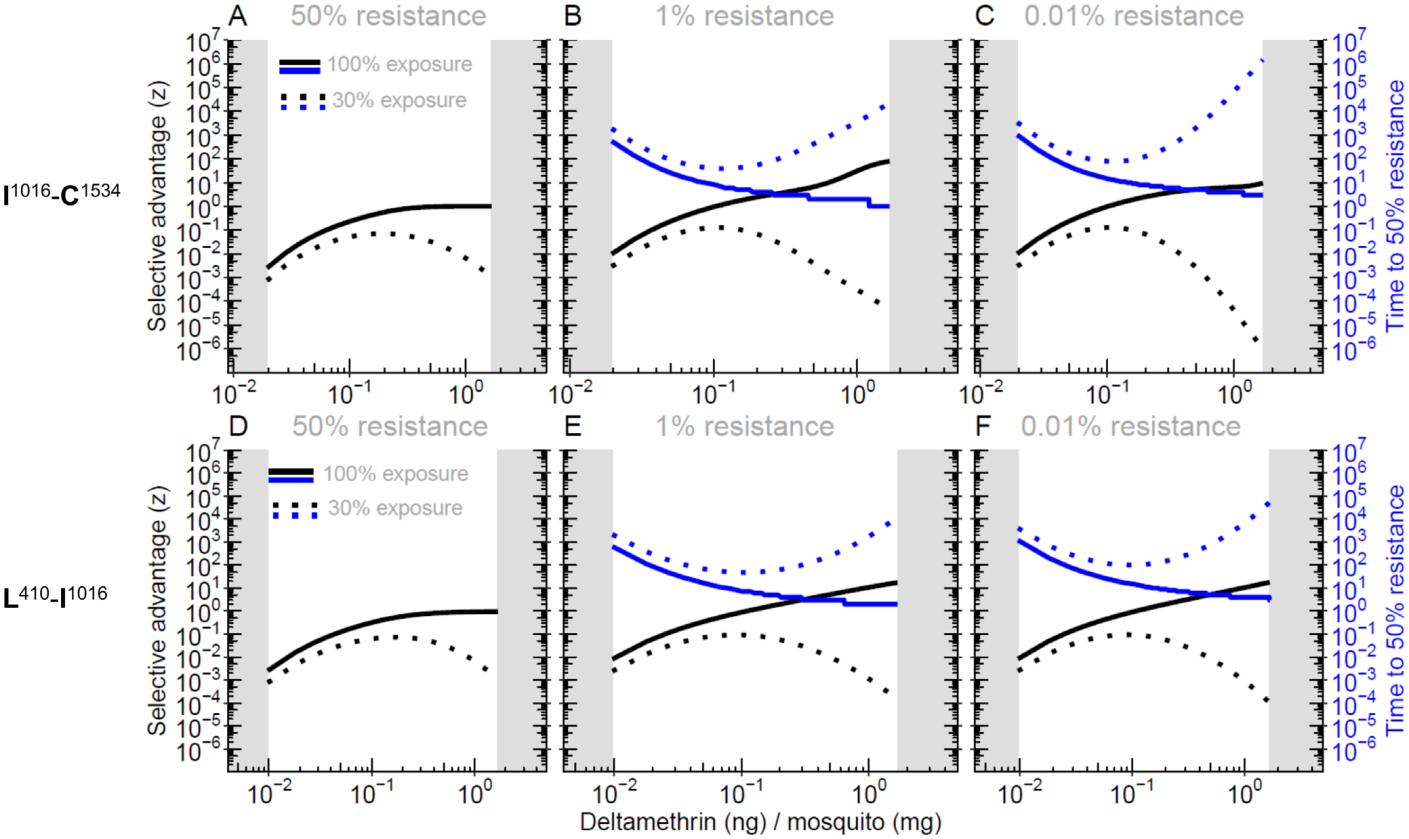
The relationship between the selective advantage (*z*) and time to 50% resistance (T_50_) within the windows of selection (WoS). Plots A‐C represent the WoS of **LL**‐VV‐FF, **LL**‐V**I**‐F**C**, and **LL**‐**II**‐**CC** for resistant haplotype **I** ^1016^‐**C** ^1534^. Plots D‐F represent the WoS of VV‐VV‐**CC**, V**L**‐V**I**‐**CC**, and **LL**‐**II**‐**CC** for resistant haplotype **L** ^410^‐**I** ^1016^. Areas outside the WoS are colored gray. The starting population for all calculations was assumed to be in Hardy–Weinberg equilibrium with the resistant haplotype frequency starting at 50% for plots A and D, 1% for plots B and E, and 0.01% for plots C and F. The x‐axis shows the amount of deltamethrin (ng) per mosquito (mg) on a log scale. The primary y‐axis (black) shows the selective advantage (z) on a log scale, calculated following equation (3). The secondary y‐axis (blue) shows the time (generations) to 50% resistance on a log scale. Plots A and D lack a time to 50% resistance line because their starting populations are already at 50% resistance. The selective advantage and time to 50% resistance are shown for 100% insecticide exposure (solid lines) and 30% insecticide exposure (dotted lines) scenarios.

For the **I** ^1016^‐**C** ^1534^ haplotype, the WoS ranged from 0.020 to 1.7 ng deltamethrin per mg mosquito (Figure 4A–C). The WoS for the **L** ^410^‐**I** ^1016^ haplotype ranged from 0.0010 to 1.7 ng deltamethrin per mg mosquito (Figure 4D–F). The selective advantage (z) and time to 50% resistant haplotype frequency ($T_{50}$) values followed the same patterns for both haplotypes. Using mathematical simulations, we found that when all mosquitoes were exposed to insecticides, the selective advantage was greatest at the highest doses in the WoS and declined as dosages decreased, regardless of the starting haplotype frequency (50%, 1%, or 0.01%, Figure 4). Consequently, $T_{50}$ was shortest at high dosages and increased as dosages approached the lower boundary of the selection window. Interestingly, a different pattern was observed when insecticide exposure was simulated to be only 30% of the population. Here, the selective advantage peaked at intermediate dosages and was lowest at the high and low ends of the WoS. $T_{50}$ was, therefore, the shortest at these intermediate dosages but still remained greater than the equivalent simulations with 100% insecticide exposure. Specifically, at the highest level of selection within the WoS and with a starting resistance haplotype frequency of 1% (Figure 4B,E), reducing exposure from 100% to 30% led to a 40‐fold and 24‐fold increase in $T_{50}$ for the **I** ^1016^‐**C** ^1534^ and **L** ^410^‐**I** ^1016^ haplotypes, respectively. Similarly, at the highest level of selection and with a starting resistance haplotype frequency of 0.01% (Figure 4C,F), the same reduction in exposure (100% to 30%) led to a 22.6‐fold and 33.3‐fold increase in $T_{50}$ for the **I** ^1016^‐**C** ^1534^ and **L** ^410^‐**I** ^1016^ haplotypes, respectively. These general patterns stayed consistent even when these haplotypes were assumed to be fully recessive (Figure S2) or fully dominant (Figure S3). Additionally, $T_{50}$ varied with the level of dominance, with fully recessive haplotypes requiring more generations to reach 50% resistance than fully dominant haplotypes, particularly under 30% exposure conditions. Specifically, at the highest levels of selection for both resistant haplotypes, full recessiveness required 15‐fold and 833‐fold more generations than full dominance to reach $T_{50}$ under 30% exposure, for the 1% and 0.01% starting resistance frequencies, respectively.

## 4 Discussion

In this study, we extensively analyzed the selective pressures on emerging and common *kdr* mutations in *Ae. aegypti* mosquitoes, creating highly detailed deltamethrin dose–response curves of genetic crosses and performing mathematical simulations to assess the rate of resistance evolution under various treatment strategies. The triple mutant haplotype **L**‐**I**‐**C** was correlated with the highest levels of resistance; resistant haplotypes **I** ^1016^‐**C** ^1534^ and **L** ^410^‐**I** ^1016^ were shown to be incompletely recessive with some evidence of epistatic interactions between loci. The incompletely recessive inheritance patterns observed here may also reflect the influence of additional genetic background effects beyond the focal *kdr* loci. Within the windows of selection, the selective advantage depended on the insecticide dosage, the insecticide exposure level experienced by the mosquito population, and the dominance level of each haplotype. These findings highlight the complexity of natural selection in mosquito populations and the challenges in creating straightforward guidelines for managing insecticide resistance in vector control.

There has been a long‐standing debate about whether xenobiotic resistance evolves more rapidly under higher or lower dosages (Drlica and Zhao 2007; Helps et al. 2017; Kouyos et al. 2014; Negri et al. 2000). Current research in this field has produced mixed results, with some studies suggesting that higher dosages accelerate resistance evolution (Gatenby et al. 2009; Helps et al. 2017; Huijben et al. 2013), while it is also shown that, under certain conditions, lower dosages may actually promote resistance evolution (Helps et al. 2017). Specifically, lower dosages may promote resistance evolution through increased survival of heterozygotes and/or individuals carrying partially resistant alleles, while higher dosages may impose stronger selection for highly resistant alleles. Our findings show that this relationship between dosage and the strength of selection for *kdr* mutations is more complex than previously thought. The dose at which the selective advantage for resistant mutants is highest depends on various factors, with treatment coverage being particularly critical. A key aspect of the debate over optimal dosages involves the role of fitness costs associated with resistance. Studies on other organisms, such as malaria parasites and cancer cells, have shown that higher dosages can intensify selection for resistance because they eliminate the competitive suppression of less fit resistant strains by more fit susceptible ones in the absence of treatment (Huijben et al. 2013). Importantly, our simulations did not account for competitive interactions or fitness differences between genotypes and showed that high deltamethrin dosages can confer the greatest selective advantage for *kdr* mutations under certain conditions, even without the effect of competitive release. Because fitness costs may substantially alter resistance spread and competitive dynamics, assessing genotype‐specific fitness costs and incorporating these parameters into future simulations would provide a more comprehensive understanding of how selection pressures across different insecticide dosages shape evolutionary dynamics.

The selective advantage within the WoS and at the upper and lower bounds of the WoS identify the intensity of selection that occurs at various insecticide dosages, but translating these findings to real‐world applications poses challenges. This difficulty arises because the dose–response curves in our study were derived from an artificial method of insecticide exposure, where insecticide was applied directly to the ventral thorax of a cold‐knocked‐down mosquito under otherwise optimal lab conditions. Although this gold standard method yields the most accurate dose–response curves (Althoff and Huijben 2022; World Health Organization 2009), the absolute dose values cannot be directly extrapolated to natural exposure scenarios (discussed in Namias et al. 2021). Similarly, these controlled laboratory conditions do not capture the full ecological complexity influencing resistance evolution in field populations. In addition, because this study focused on a single insecticide, the quantitative patterns observed here may not necessarily extend to other insecticides or insecticide classes. However, qualitative patterns of response are likely to hold true across different exposure methods, such as aerial fogging, treated bed nets, residual sprays, or larviciding (e.g., lab‐exposure and field‐exposure response trends align (Estep et al. 2025)). Despite these limitations in direct translation to field dosages, we contend that the WoS framework can be a valuable tool for developing evidence‐based resistance management strategies. A critical consideration in public health insecticide use is the inevitable decline of insecticide concentrations in the environment over time. Without reapplication, environmental insecticide dosages will pass through the WoS from higher to lower levels, a factor that must be accounted for in resistance management design. Our simulations reveal that maximum selection occurs at lower dosages when coverage levels were at 30% compared to full coverage. Refuge strategies, commonly used in agriculture for resistance management, are designed to intentionally reduce insecticide coverage and maintain susceptible alleles through migration from untreated areas (reviewed in Estep et al. 2023; Grossman et al. 2018). The impact of coverage level is thus important to consider when refuge strategies are incorporated. However, while it is generally acknowledged that realized insecticide coverage levels after operational application are less than 100%, due to suboptimal application and unintended or natural refuge areas, data on more realistic insecticide coverage levels are absent. Gaining insight into these levels of coverage and the dosages mosquitoes encounter is crucial for designing effective resistance management strategies. If coverage is low or refuge strategies are applied, our findings suggest that selection for *kdr* mutations and other recessive resistance mechanisms may be lowest under either high, sustained concentrations of insecticides in the environment or at significantly lower concentrations. For dominantly inherited resistance these findings would likely differ, as resistance selection is influenced by inheritance patterns.

The absence of dose–response data for heterozygote mosquitoes has been hindering the development of the WoS framework for mosquitoes (South et al. 2020). The *kdr* genotypes included in our study represent the six most common genotypes observed in Western hemisphere populations with respect to loci 1016 and 1534 (Estep et al. 2018, 2023; Grossman et al. 2018; Hernandez et al. 2021; Mack et al. 2021; Vera‐Maloof et al. 2015, 2020; Wimmer et al. 2025). However, because these experimentally characterized genotypes are not isogenic, minor differences in broader genetic background, despite their shared geographic origin, may also contribute to phenotypic variation and influence simulation outcomes. The strong selective advantage shown for both resistant haplotypes **I** ^1016^‐**C** ^1534^ and **L** ^410^‐**I** ^1016^ within theoretical deltamethrin‐exposed environments provides a baseline explanation for the high frequency of the **I** ^1016^‐**C** ^1534^ haplotype in several locations on a known 410 background (Wimmer et al. 2025) and on unknown 410 backgrounds (Estep et al. 2018, 2023; Grossman et al. 2018; Hernandez et al. 2021; Vera‐Maloof et al. 2015, 2020). The accuracy of resistance data is crucial for the reliable development of the WoS framework. Our findings demonstrate that resistance levels are highly influenced by how the data is normalized. In standard fixed‐dose bioassays, doses are typically standardized for female mosquitoes. This practice arises partly from the assumption that males are more susceptible (Boubidi et al. 2016), but primarily because quantitative bioassays are frequently performed only on female mosquitoes due to their significance in public health. In our study, males indeed exhibited higher mortality rates than females across dosages. However, weight adjustment largely eliminated this difference (Figure 2). The negligible resistance differences between sexes following weight correction highlight the significant impact of mosquito body mass on resistance levels. Given the common occurrence of weight variations among mosquitoes, often due to climatic conditions or environment (reviewed in Namias et al. 2021; World Health Organization 2009), the absence of weight correction in most insecticide‐resistance bioassays likely introduces considerable noise in the data, reducing the ability to distinguish between resistant and susceptible populations.

This study represents a first step towards evidence‐based resistance management and underscores the need for additional data to refine these strategies. By focusing on experimentally characterized *kdr* genotypes under controlled laboratory conditions, we were able to isolate the contribution of specific target‐site mutations to selection dynamics across insecticide dosages. Similar studies across additional insecticides, resistance mechanisms, exposure scenarios, and vector species will be needed to develop a more comprehensive understanding of resistance evolution in natural populations. Such data can be used to formulate mathematical models capable of predicting the trajectory of insecticide resistance evolution across varied exposure dosages, paving the way for innovative, evidence‐driven resistance management strategies as well as predicting the impact of insecticide exposures from household and agricultural use (Pokhrel and Ottea 2023). Having such data for multiple insecticides would allow for the development of an overall model that incorporates resistance management strategies such as insecticidal mixtures, rotations, or mosaics (Madgwick and Kanitz 2024; South et al. 2020). Vector‐borne diseases kill more than 700,000 people annually (World Health Organization 2024), with no sign of improvement in recent decades. While many factors are involved in these concerning trends, insecticide resistance is an important contributor (Alout et al. 2017; Kleinschmidt et al. 2018; Tokponnon et al. 2019). The introduction of novel insecticides for public health occurs at an agonizingly slow pace, making it critical to preserve the effectiveness of these new chemicals to sustain our capacity to combat vector‐borne diseases. While insecticide surveillance data is valuable, it alone cannot prevent resistance. The priority must be to gather more comprehensive data that elucidates the evolutionary pressures driving the persistence and spread of resistant mutants. This deeper understanding will be essential for developing evidence‐based strategies to effectively manage and mitigate insecticide resistance.

## Funding

This work was supported by the National Science Foundation (2047572, 2052363) and the USDA National Program‐104 (CRIS: 6036‐32000‐052‐000D, MTRA: 58‐6036‐1‐0010).

## Conflicts of Interest

The authors declare no conflicts of interest.

## Supporting information


**Table S1:** *Ae. aegypti* genotypes included in this study with their genotype for three *kdr* mutations (V410L, V1016I and F1534C respectively) and the corresponding parental genotype(s). All parental genotypes, except for Rockefeller (ROCK), were isolated from St. Augustine, Florida (2016). Genetic crosses between isolates are indicated by an ‘x’, showing the combination of the parental strains used for females (♀) and males (♂).
**Table S2:** Goodness‐of‐fit parameters for all probit models used in this study.
**Table S3:** Lethal dose of deltamethrin (ng per mg mosquito mass) to kill 50% (LD50) with 95% confidence intervals [95% CI] determined by a quasibinomial probit link glm of the dose–response data points (n) that contained mortality greater than 0% and less than 100%. LD50 and 95% CI are displayed for sex separately. Wald ratio tests with Bonferroni‐adjusted *p*‐values were used to determine significant differences between sexes of the same.
**Figure S1:** The degree of dominance (DoD) across all possible lethal doses for (A) the **I** ^1016^‐**C** ^1534^ haplotype (based of genotypes **LL**‐VV‐FF, **LL**‐V**I**‐F**C**, and **LL**‐**II**‐**CC**; solid blue line), and (B) the **L** ^410^‐**I** ^1016^ haplotype (based on genotypes VV‐VV‐**CC**, V**L**‐V**I**‐**CC**, **LL**‐**II**‐**CC**; dotted red line). The shaded regions represent the 95% confidence interval of the DoD, calculated from the confidence intervals of the lethal doses. The lethal dose causing 50% mortality (LD_50_) is identified by a vertical, dashed, black line (where most degree of dominance calculations occur).
**Figure S2:** The relationship between the selective advantage and time to 50% resistance within the windows of selection (WoS), assuming full recessiveness of the haplotypes of interest. Plots A‐C represent the WoS of **LL**‐VV‐FF, **LL**‐V**I**‐F**C**, and **LL**‐**II**‐**CC** for haplotype **I** ^1016^‐**C** ^1534^. Plots D‐F represent the WoS of VV‐VV‐**CC**, V**L**‐V**I**‐**CC**, and **LL**‐**II**‐**CC** for haplotype **L** ^410^‐**I** ^1016^. Areas outside the windows of selection are colored gray. The starting population for all calculations was assumed to be in Hardy–Weinberg equilibrium with the resistant haplotype frequency starting at 50% for plots A and D, 1% for plots B and E, and 0.01% for plots C and F. The x‐axis shows the amount of deltamethrin (ng) per mosquito (mg) on a log scale. The primary y‐axis (black) shows the selective advantage (z) on a log scale, calculated following equation (3). The secondary y‐axis (blue) shows the time (generations) to 50% resistance on a log scale. Plots A and D lack a time to 50% resistance line because their starting populations are already at 50% resistance. The selective advantage and time to 50% resistance are shown for 100% insecticide exposure (solid lines) and 30% insecticide exposure (dotted lines) scenarios.
**Figure S3:** The relationship between the selective advantage and time to 50% resistance within the windows of selection (WoS), assuming full dominance of the haplotypes of interest. Plots A‐C represent the WoS of **LL**‐VV‐FF, **LL**‐V**I**‐F**C**, and **LL**‐**II**‐**CC** for haplotype **I** ^1016^‐**C** ^1534^. Plots D‐F represent the WoS of VV‐VV‐**CC**, V**L**‐V**I**‐**CC**, and **LL**‐**II**‐**CC** for haplotype **L** ^410^‐**I** ^1016^. Areas outside the windows of selection are colored gray. The starting population for all calculations was assumed to be in Hardy–Weinberg equilibrium with the resistant haplotype frequency starting at 50% for plots A and D, 1% for plots B and E, and 0.01% for plots C and F. The x‐axis shows the amount of deltamethrin (ng) per mosquito (mg) on a log scale. The primary y‐axis (black) shows the selective advantage (z) on a log scale, calculated following equation (3). The secondary y‐axis (blue) shows the time (generations) to 50% resistance on a log scale. Plots A and D lack a time to 50% resistance line because their starting populations are already at 50% resistance. The selective advantage and time to 50% resistance are shown for 100% insecticide exposure (solid lines) and 30% insecticide exposure (dotted lines) scenarios.

## Data Availability

The data that support the findings of this study are openly available in ASU Research Data Repository at https://doi.org/10.48349/ASU/NEQQOC.
