# Supplementary material for: The Evolution of Knockdown Resistance: Genotypic and Phenotypic Insights Into Aedes aegypti Insecticide Resistance
Source: Evol Appl. 2026 Jul 23;19(7):e70305. doi: 10.1111/eva.70305 (PMC13392631; doi:10.1111/eva.70305)
Supplement: Supplementary file 1 — Table S1: Ae. aegypti genotypes included in this study with their genotype for three kdr mutations (V410L, V1016I and F1534C respectively) and the corresponding parental genotype(s). All parental genotypes, except for Rockefeller (ROCK), were isolated from St. Augustine, Florida (2016). Genetic crosses between isolates are indicated by an ‘x’, showing the combination of the parental strains used for females (♀) and males (♂). Table S2: Goodness‐of‐fit parameters for all probit models used in this study. Table S3: Lethal dose of deltamethrin (ng per mg mosquito mass) to kill 50% (LD50) with 95% confidence intervals [95% CI] determined by a quasibinomial probit link glm of the dose–response data points (n) that contained mortality greater than 0% and less than 100%. LD50 and 95% CI are displayed for sex separately. Wald ratio tests with Bonferroni‐adjusted p‐values were used to determine significant differences between sexes of the same. Figure S1: The degree of dominance (DoD) across all possible lethal doses for (A) the I 1016‐C 1534 haplotype (based of genotypes LL‐VV‐FF, LL‐VI‐FC, and LL‐II‐CC; solid blue line), and (B) the L 410‐I 1016 haplotype (based on genotypes VV‐VV‐CC, VL‐VI‐CC, LL‐II‐CC; dotted red line). The shaded regions represent the 95% confidence interval of the DoD, calculated from the confidence intervals of the lethal doses. The lethal dose causing 50% mortality (LD50) is identified by a vertical, dashed, black line (where most degree of dominance calculations occur). Figure S2: The relationship between the selective advantage and time to 50% resistance within the windows of selection (WoS), assuming full recessiveness of the haplotypes of interest. Plots A‐C represent the WoS of LL‐VV‐FF, LL‐VI‐FC, and LL‐II‐CC for haplotype I 1016‐C 1534. Plots D‐F represent the WoS of VV‐VV‐CC, VL‐VI‐CC, and LL‐II‐CC for haplotype L 410‐I 1016. Areas outside the windows of selection are colored gray. The starting population for all calculations was assumed to b [file EVA-19-e70305-s001.docx]

**Supplementary Tables and Figures**

**Supplementary Table S1:** *Ae. aegypti* genotypes included in this study with their genotype for three *kdr* mutations (V410L, V1016I and F1534C respectively) and the corresponding parental genotype(s). All parental genotypes, except for Rockefeller (ROCK), were isolated from St. Augustine, Florida (2016). Genetic crosses between isolates are indicated by an ‘x’, showing the combination of the parental strains used for females (**♀**) and males (♂).

| Genotype | 410 locus | 1016 locus | 1534 locus | Total *kdr* mutations | Parental genotype(s) |
| --- | --- | --- | --- | --- | --- |
| ROCK  (VV-VV-FF) | VV | VV | FF | 0 | ROCK |
| VV-VV-**CC** | VV | VV | CC | 2 | VV-VV-**CC** |
| V**L**-VV-F**C** | VL | VV | FC | 2 | **LL**-VV-FF (**♀**) x VV-VV-**CC** (♂) |
| **LL**-VV-FF | LL | VV | FF | 2 | **LL**-VV-FF |
| V**L**-V**I**-**CC** | VL | VI | CC | 4 | **LL**-**II**-**CC** (**♀**) x VV-VV-**CC** (♂) |
| **LL**-V**I**-F**C** | LL | VI | FC | 4 | **LL**-**II**-**CC** (**♀**) x **LL**-VV-FF (♂) |
| **LL**-**II**-**CC** | LL | II | CC | 6 | **LL**-**II**-**CC** |

**Supplementary Table S2:** Goodness-of-fit parameters for all probit models used in this study.

| **Genotype** | **Weight correction status** | **Sex** | **Residual deviance** | **Residual degrees of freedom** | **Dispersion** |
| --- | --- | --- | --- | --- | --- |
| ROCK  (VV-VV-FF) | Yes | Both | 3.567 | 41 | 0.135 |
|  |  | Female | 1.87 | 19 | 0.133 |
|  |  | Male | 1.058 | 20 | 0.169 |
|  | No | Female | 2.089 | 19 | 0.15 |
|  |  | Male | 1.11 | 20 | 0.155 |
| VV-VV-**CC** | Yes | Both | 5.159 | 37 | 0.141 |
|  |  | Female | 3.154 | 19 | 0.16 |
|  |  | Male | 1.481 | 16 | 0.199 |
|  | No | Female | 4.921 | 19 | 0.241 |
|  |  | Male | 2.259 | 16 | 0.26 |
| V**L**-VV-F**C** | Yes | Both | 4.215 | 52 | 0.1 |
|  |  | Female | 1.704 | 26 | 0.069 |
|  |  | Male | 1.895 | 24 | 0.121 |
|  | No | Female | 1.978 | 26 | 0.074 |
|  |  | Male | 2.113 | 24 | 0.128 |
| **LL**-VV-FF | Yes | Both | 3.08 | 31 | 0.105 |
|  |  | Female | 0.515 | 12 | 0.048 |
|  |  | Male | 2.228 | 17 | 0.131 |
|  | No | Female | 1.233 | 12 | 0.099 |
|  |  | Male | 2.256 | 17 | 0.14 |
| V**L**-V**I**-**CC** | Yes | Both | 4.772 | 57 | 0.082 |
|  |  | Female | 3.141 | 29 | 0.106 |
|  |  | Male | 1.219 | 26 | 0.046 |
|  | No | Female | 3.105 | 29 | 0.104 |
|  |  | Male | 1.897 | 26 | 0.072 |
| **LL**-V**I**-F**C** | Yes | Both | 2.411 | 31 | 0.072 |
|  |  | Female | 0.681 | 15 | 0.044 |
|  |  | Male | 1.583 | 14 | 0.103 |
|  | No | Female | 1.175 | 15 | 0.076 |
|  |  | Male | 1.666 | 14 | 0.11 |
| **LL**-**II**-**CC** | Yes | Both | 3.588 | 31 | 0.114 |
|  |  | Female | 1.216 | 14 | 0.089 |
|  |  | Male | 2.096 | 15 | 0.172 |
|  | No | Female | 1.481 | 14 | 0.108 |
|  |  | Male | 1.885 | 15 | 0.222 |

**Supplementary Table S3:** Lethal dose of deltamethrin (ng per mg mosquito mass) to kill 50% (LD_50_) with 95% confidence intervals [95%CI] determined by a quasibinomial probit link glm of the dose-response data points (n) that contained mortality greater than 0% and less than 100%. LD_50_ and 95%CI are displayed for sex separately. Wald ratio tests with Bonferroni-adjusted p-values were used to determine significant differences between sexes of the same.

| **Genotype** | **Females** | |  | **Males** | |  | **Wald ratio test z value** | **p-adj** |
| --- | --- | --- | --- | --- | --- | --- | --- | --- |
|  | **n** | **LD_50_ [95%CI]** |  | **n** | **LD_50_ [95%CI]** |  |  |  |
| ROCK  (VV-VV-FF) | 21 | 0.008 [0.007 – 0.010] |  | 19 | 0.006 [0.004 – 0.008] |  | 2.03 | 0.295 |
| VV-VV-**CC** | 21 | 0.062 [0.050 – 0.078] |  | 18 | 0.055 [0.046 – 0.066] |  | 0.84 | 1.000 |
| V**L**-VV-F**C** | 28 | 0.081 [0.073 – 0.090] |  | 26 | 0.066 [0.059 – 0.075] |  | 2.51 | 0.085 |
| **LL**-VV-FF | 15 | 0.080 [0.073 – 0.087] |  | 19 | 0.082 [0.070 – 0.096] |  | -0.33 | 1.000 |
| V**L**-V**I**-**CC** | 32 | 0.097 [0.084 – 0.111] |  | 28 | 0.108 [0.101 – 0.117] |  | -1.40 | 1.000 |
| **LL**-V**I**-F**C** | 16 | 0.139 [0.128 – 0.151] |  | 15 | 0.129 [0.114 – 0.146] |  | 0.99 | 1.000 |
| **LL**-**II**-**CC** | 16 | 0.398 [0.346 – 0.458] |  | 19 | 0.338 [0.288 – 0.398] |  | 1.45 | 0.954 |


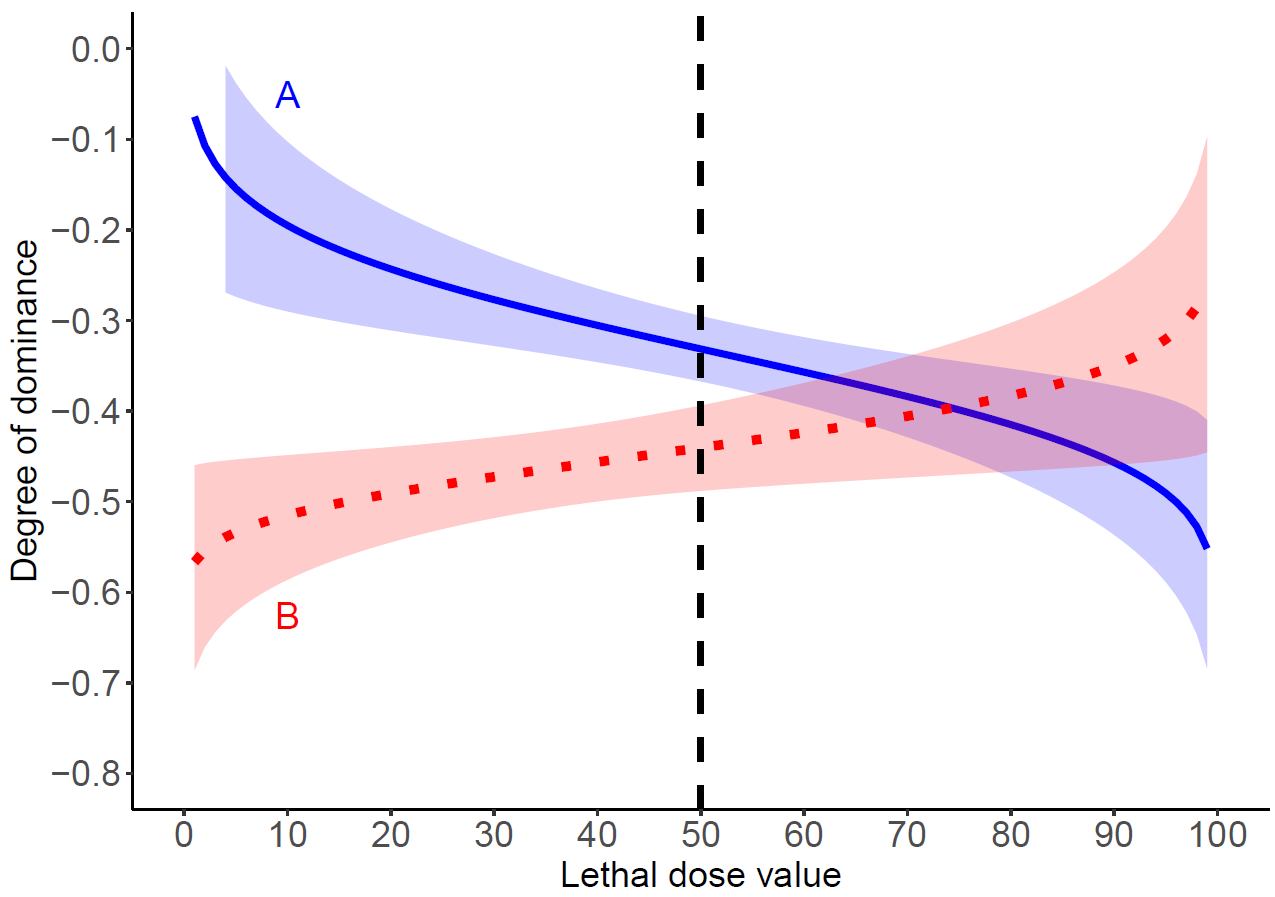


**Supplementary Figure S1**. The degree of dominance (DoD) across all possible lethal doses for (A) the **I**^1016^-**C**^1534^ haplotype (based of genotypes **LL**-VV-FF, **LL**-V**I**-F**C**, and **LL**-**II**-**CC**; solid blue line), and (B) the **L**^410^-**I**^1016^ haplotype (based on genotypes VV-VV-**CC**, V**L**-V**I**-**CC**, **LL**-**II**-**CC**; dotted red line). The shaded regions represent the 95% confidence interval of the DoD, calculated from the confidence intervals of the lethal doses. The lethal dose causing 50% mortality (LD_50_) is identified by a vertical, dashed, black line (where most degree of dominance calculations occur).


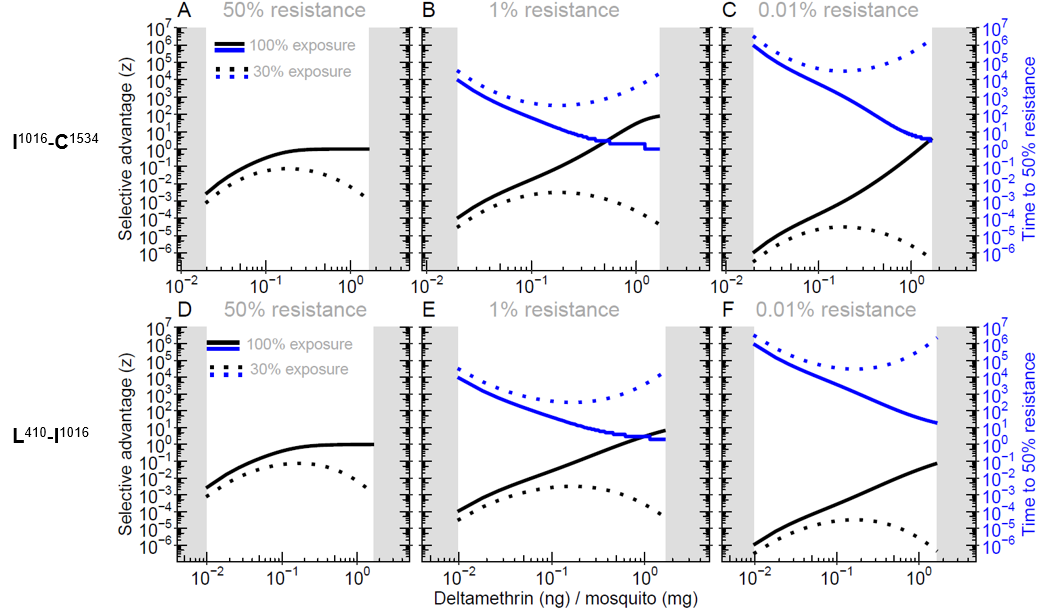


**Supplementary Figure S2**. The relationship between the selective advantage and time to 50% resistance within the windows of selection (WoS), assuming full recessiveness of the haplotypes of interest. Plots A-C represent the WoS of **LL**-VV-FF, **LL-**V**I-**F**C**, and **LL-II-CC** for haplotype **I**^1016^-**C**^1534^. Plots D-F represent the WoS of VV-VV-**CC**, V**L**-V**I**-**CC**, and **LL-II-CC** for haplotype **L**^410^-**I**^1016^. Areas outside the windows of selection are colored gray. The starting population for all calculations was assumed to be in Hardy-Weinberg equilibrium with the resistant haplotype frequency starting at 50% for plots A and D, 1% for plots B and E, and 0.01% for plots C and F. The X-axis shows the amount of deltamethrin (ng) per mosquito (mg) on a log scale. The primary y-axis (black) shows the selective advantage (z) on a log scale, calculated following equation 3. The secondary y-axis (blue) shows the time (generations) to 50% resistance on a log scale. Plots A and D lack a time to 50% resistance line because their starting populations are already at 50% resistance. The selective advantage and time to 50% resistance are shown for 100% insecticide exposure (solid lines) and 30% insecticide exposure (dotted lines) scenarios.


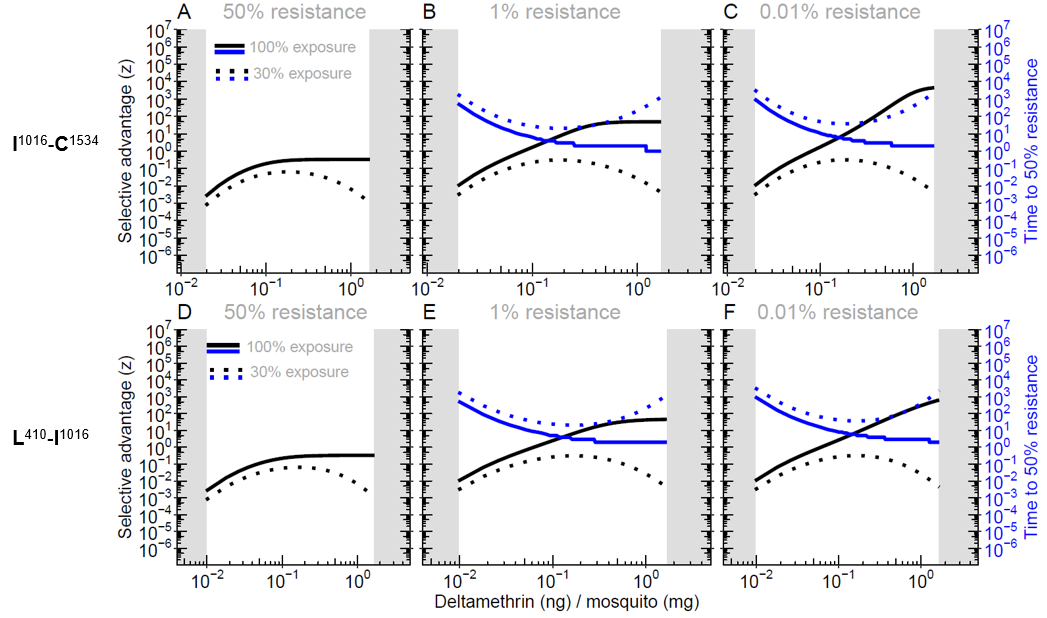


**Supplementary Figure S3**. The relationship between the selective advantage and time to 50% resistance within the windows of selection (WoS), assuming full dominance of the haplotypes of interest. Plots A-C represent the WoS of **LL**-VV-FF, **LL-**V**I-**F**C**, and **LL-II-CC** for haplotype **I**^1016^-**C**^1534^. Plots D-F represent the WoS of VV-VV-**CC**, V**L**-V**I**-**CC**, and **LL-II-CC** for haplotype **L**^410^-**I**^1016^. Areas outside the windows of selection are colored gray. The starting population for all calculations was assumed to be in Hardy-Weinberg equilibrium with the resistant haplotype frequency starting at 50% for plots A and D, 1% for plots B and E, and 0.01% for plots C and F. The X-axis shows the amount of deltamethrin (ng) per mosquito (mg) on a log scale. The primary y-axis (black) shows the selective advantage (z) on a log scale, calculated following equation 3. The secondary y-axis (blue) shows the time (generations) to 50% resistance on a log scale. Plots A and D lack a time to 50% resistance line because their starting populations are already at 50% resistance. The selective advantage and time to 50% resistance are shown for 100% insecticide exposure (solid lines) and 30% insecticide exposure (dotted lines) scenarios.
